# The Relationship between *ACE*, *ACTN3* and *MCT1* Genetic Polymorphisms and Athletic Performance in Elite Rugby Union Players: A Preliminary Study

**DOI:** 10.3390/genes13060969

**Published:** 2022-05-28

**Authors:** Massimo Pasqualetti, Maria Elisabetta Onori, Giulia Canu, Giacomo Moretti, Angelo Minucci, Silvia Baroni, Alvaro Mordente, Andrea Urbani, Christel Galvani

**Affiliations:** 1 UOC di Chimica Biochimica e Biologia Molecolare Clinica, Fondazione Policlinico Universitario A. Gemelli I.R.C.C.S., Via della Pineta Sacchetti 217, 00168 Rome, Italy; massimo.pasqualetti@yahoo.com (M.P.); mariaelisabetta.onori@guest.policlinicogemelli.it (M.E.O.); giuliacanu@gmail.com (G.C.); giacomo.moretti@policlinicogemelli.it (G.M.); angelo.minucci@policlinicogemelli.it (A.M.); silvia.baroni@policlinicogemelli.it (S.B.); andrea.urbani@policlinicogemelli.it (A.U.); 2 Dipartimento di Scienze Biotecnologiche di Base, Cliniche Intensivologiche e Perioperatorie, Università Cattolica del Sacro Cuore, Largo Francesco Vito 1, 00168 Rome, Italy; alvaro.mordente@unicatt.it; 3 Dipartimento di Scienze di Laboratorio e Infettivologiche, Fondazione Policlinico Universitario A. Gemelli I.R.C.C.S., Via della Pineta Sacchetti 217, 00168 Rome, Italy; 4 Laboratorio di Scienze Dell’esercizio Fisico e Dello Sport, Dipartimento di Psicologia, Università Cattolica del Sacro Cuore, Viale Suzzani 279, 20162 Milan, Italy

**Keywords:** rugby union, genomics, *ACE*, *ACTN3*, *MCT1*, athletic performance

## Abstract

Athletic performance is influenced by many factors such as the environment, diet, training and endurance or speed in physical effort and by genetic predisposition. Just a few studies have analyzed the impact of genotypes on physical performance in rugby. The aim of this study was to verify the modulation of genetic influence on rugby-specific physical performance. Twenty-seven elite rugby union players were involved in the study during the in-season phase. Molecular genotyping was performed for: angiotensin-converting enzyme (*ACE* rs4646994), alfa-actinin-3 (*ACTN3* rs1815739) and monocarboxylate transporter 1 (*MCT1* rs1049434) and their variants. Lean mass index (from skinfolds), lower-limb explosive power (countermovement jump), agility (505), speed (20 m), maximal aerobic power (Yo-yo intermittent recovery test level 1) and repeated sprint ability (12 × 20 m) were evaluated. In our rugby union players *ACE* and *ACTN3* variants did not show any influence on athletic performance. *MCT1* analysis showed that TT-variant players had the highest peak vertical power (*p* = 0.037) while the ones with the AA genotype were the fastest in both agility and sprint tests (*p* = 0.006 and *p* = 0.012, respectively). Considering the T-dominant model, the AA genotype remains the fastest in both tests (agility: *p* = 0.013, speed: *p* = 0.017). Only the *MCT1* rs1049434 A allele seems to be advantageous for elite rugby union players, particularly when power and speed are required.

## 1. Introduction

Athletic performances are influenced by environmental factors (training, lifestyle, physiological and psychological factors) and genetics [1]. On average, 66% (depending on sports discipline) of the variance in the athlete status is explained by genetic factors [2]. The combination of phenotype with genotype could be the key to excellence [3].

Sports genetics relates to the genotypic basis of sport phenotype. Angiotensin-converting enzyme (*ACE*) and alfa-actinin 3 (*ACTN3*) are two of the most studied genes influencing endurance, strength/power and other phenotypic traits concerning athletic performance [2,4,5], while monocarboxylate transporter 1 (*MCT1*) gene analysis, instead, showed correlations with skeletal muscles capacity to take up lactate from the circulation [6] which is linked with elite sprint/power athletic status [7].

In particular, the *ACE* is the major enzyme produced in the renin-angiotensin system. It is considered by Woods [8] not a “gene for human performance, but a marker of modulation”. Among others, *ACE* is associated with endurance polygenic [2,4,5,8]. Generally, the I allele is more frequent in elite endurance athletes, while the D allele among those engaged in more power-oriented sports, given the enzyme highest fast-twitch muscle fibers [2,4,5,8]. *ACTN3* is known as the “speed gene” [9] and more recently it has been investigated with its association with physical performance. In humans, *ACTN3* is expressed only in type II muscle fibers (fast twitch). The R allele was found more frequent in speed, power and strength athletes than non-athletes [2,4,5,10], while it seems that the XX genotype tends to be associated with endurance [2,5,10]. *MCT1* has been found particularly in oxidative muscle fibers with high mitochondrial content (type I—slow twitch). Fedotovskaya et al. [11] reported that A allele and AA genotype were higher in endurance-oriented rowers compared with the control group, and also the presence of T allele identified a higher blood lactate level during high-intensity circuit training [12]. Other researchers suggested that the TT genotype characterized the elite sprint/power athletic status in Polish [7] and Brazilian [13] athletes.

Rugby union (RU) is an intermittent effort field-based team sport with a high frequency of contact. Rugby is primarily an anaerobic sport, although the aerobic system is utilized during rest period to replenish energy stores [14]. During a match, players perform high-intensity movements of relatively short duration, followed by a short resting period and covered distances range from ≈4000 m to ≈7000 m [15]. Each of the two teams presents 15 players on field, divided into playing position: forwards and backs. Brazier et al. [16] identified key anthropometric (low body fat percentage) and physiological (well-developed speed, agility, lower-body power and strength) properties required for elite performance in RU. From an anthropometric point of view elite athletes are heavier with lower skinfold thicknesses and higher lean mass index (LMI) than amateur and semi-professionals; from a physiological perspective they are stronger, faster and more powerful. Likewise, maximal aerobic and anaerobic powers are very important but they are not recognized as key discriminators [16]. Every playing position requires specific physical demands [14]. Forwards are involved in scrums, pushing and lifting during the lineouts, play more contact phases, run shorter distances and have a lower work to rest ratio. Instead, the backs, due to their ability to play on wider spaces, run longer distances than forwards, play less contact phases and have a higher work to rest ratio [17,18]. About their physicality, forwards are typically heavier and stronger than backs; while backs are faster and more agile than forwards [19]. Furthermore, backs tend to be leaner, shorter and more aerobically fit relative to body mass and possess more explosive power than forwards [14].

In RU the majority of scientific investigation into player performance has focused on environmental factors such as training methodologies, dietary supplementation and recovery strategies [18]. All these components play a crucial role in rugby athletic development [1]. Nowadays, there is an increasing interest in studying the details that can improve the aspects of physical performance. Few studies concern RU sports genomics and its applications. Heffernan et al. [20] found no difference in *ACE* I/D genotypes between backs and forwards in elite RU athletes. He also underlined an association of *ACTN3* R577X with playing position, suggesting that an inherited fatigue resistance is more prevalent in forwards while sprinting ability in backs.

Only a few studies exist correlating both phenotype and genotype in RU [18]. Goh et al. [21] found that *ACE* I allele confers an advantage in aerobic capacity in RU players (females and males, national level). Contrarily, Bell et al. [22] reported significant differences between forwards and backs in association with ID and DD genotypes of *ACE* in some leg power elements: backs had higher values for relative force and relative power for ID genotype and they reported major displacement and velocity values for the DD genotype (males, University players) [22]. Otherwise, in a more recent study, Bell et al. [23] found no relationships between *ACTN3* genotypes and power or body composition-related phenotypes (males, University players). To the best of our knowledge, no studies investigated the association of the *MCT1* variants to RU athletes’ phenotypes.

The aim of this study was to identify a genetic correlation of *ACE* I/D, *ACTN3* R577X and *MCT1* A1470T variants with rugby-specific physiological and anthropometric variables.

## 2. Materials and Methods

### 2.1. Subjects

The entire roster of Lazio Rugby was recruited for this study (a total of 43 athletes). Using a standardized difference of 1.0, with α = 0.05 and power = 0.8, the estimated total sample size was *n* = 38 (G*Power software, ver 3.1.9.2). The injured returning to play athletes (16 players) were excluded from the study because they had not been able to undertake the phenotype tests. A total of 27 players (22.6 ± 2.9 y; 93.6 ± 16.1 kg; 180.9 ± 7.2 cm) of RU were finally enrolled. Lazio Rugby played in the TOP12 division which is the first Italian National League, so we could consider the subjects as elite players [18]. In this roster, all players were Caucasian. On average, players have at least 10 years of rugby-specific training/sports experience.

Players underwent a 2-month pre-season conditioning program with the aim to be in their peak physical conditioning at the time of testing, in November. All subjects were familiar with the entire test protocol during the pre-season, although most of the tests are part of the training routine.

The present study is part of the investigation “Anthropometric and molecular profile of professional and semiprofessional athletes”. The study protocol was approved by the Ethics Committee of the University ID 1858 and it was in accordance with Declaration of Helsinki for Human Research. The athletes were all informed of the benefits and risks of the investigation prior to signing the informed consent document to participate in the study.

### 2.2. Design

For this cross-sectional study, physical off-field tests were collected in the team’s headquarters. The tests were conducted on a last generation synthetic surface. All tests were performed by trained personnel, including medical figures and strength and conditioning coaches.

A gene candidate approach was carried out to extract genomic DNA and to determine the *ACE* I/D rs4646994, *ACTN3* rs1815739 and *MCT1* rs1049434 variants in each athlete. Genomic DNA was extracted from buccal swab sample for *ACE*, *ACTN3* and *MCT1*.

**Figure 1:**
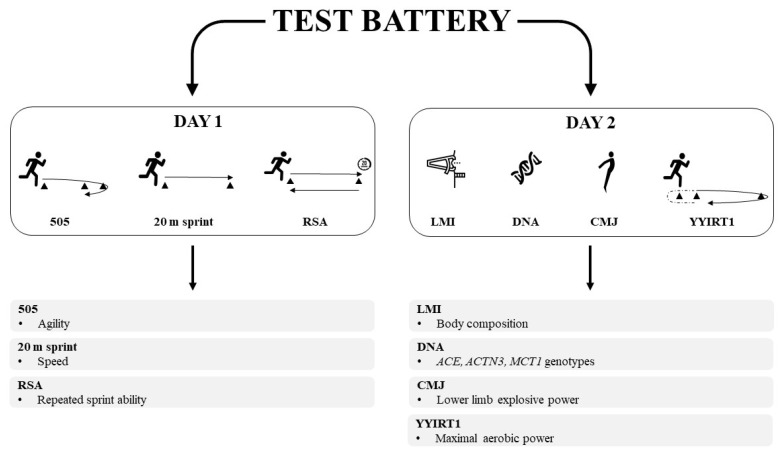
Design of the test battery.

Tests were divided into two phases: phase one in the afternoon, and the second one in the following morning. The battery of tests wanted to evaluate different features of players performance, applying some of the most used tests in RU [14]. In day 1 the athletes completed agility, speed, and repeated-sprint ability tests. During day 2, body composition, genotype analysis, lower-limb explosive power, and maximal aerobic power were assessed (Figure 1).

All players performed the tests with maximal effort. The subjects were asked to control the ingestion of alcohol and caffeine the day before and to refrain from strenuous exercise at least in the last 48 h.

### 2.3. Anthropometry and Body Composition

Height was measured using a portable stadiometer (Leicester Height Measure, Tanita Corporation, Tokyo, Japan), with a technical error of measurement of 1.5 mm and weight was measured using a digital scale (WB380P, Tanita Corporation, Tokyo, Japan) recording the body mass to the closest 0.1 kg.

To compute LMI, the sum of 7 sites skinfolds (triceps, subscapular, biceps, supraspinale, abdominal, front thigh and medial calf) was taken as value, following the ISAK procedure [24] and using Harpenden caliper (Baty International, West Sussex, UK) to 0.1 mm accuracy. LMI was then calculated as body mass/sum of 7 skinfolds^0.14^, as reported in Zemski et al. [25].

### 2.4. Physical Performance

Agility—505 test

For the 505 test, 2 timing gates (Witty, Microgate, Bolzano, Italy) were placed 5 m from a designated turning point. The players assumed a starting position 10 m from the timing gates (and therefore 15 m from the turning point). Players were instructed to accelerate as quickly as possible through the timing gates, pivot on the 15 m line, and return as quickly as possible through the timing gates. The laser of timing gates was at 1 m from the ground. Times were measured to the nearest 0.01 s with the fastest value obtained from three trials used as the 505 test score.

Speed—20 m sprint test

The sprint time was measured using dual beam electronic timing gates (Witty, Microgate, Bolzano, Italy). The laser of timing gates was at 1 m from the ground. Players were instructed to run as quickly as possible along the 20 m distance from a standing start. Two cones were placed 4 m behind the finishing line in order to maintain the maximal sprint for 24 m. The 20 m speed was measured to the nearest 0.01 s, with the fastest score from 2 trials used as the sprint score.

Repeated-sprint ability—12 × 20 m with 20 s rest cycle

Repeated-sprint ability (RSA) protocol comprised 12 sprints of 20 m distance. Players were instructed to perform each sprint with maximal effort. Each sprint was measured using dual beam electronic timing gates (Witty, Microgate, Bolzano, Italy). The laser of timing gates was at 1 m from the ground. After each sprint, the athlete came back to the end line that became the starting line 20 s after. Total sprint time was noted.

Maximal aerobic power—Yo-yo intermittent recovery test level 1

The Yo-yo intermittent recovery test level 1 (YYIRT1) consists of repeated 2 × 20 m runs back and forth between the starting, turning, and finishing line at a progressively increased speed controlled by audio beeps from a tape recorder. Between each running bout, the subjects have a 10 s active rest period, consisting of 2 × 5 m jogging. When the subjects have failed twice to reach the finishing line in time, the distance covered is recorded and represented the test result. The total duration of the test was between 6 and 20 min. The level of exhaustion was evaluated through the following three exhaustion criteria: rate of perceived exertion (RPE) ≥ 7 (using the original or the validated Italian version of Borg CR-10 scale), the lactate accumulation (La) 3 minutes after the end of the test above 8 mmol/L (Lactate Pro 2, Arkray Inc., Tokyo, Japan), and maximum heart rate (HRmax) in the last minute ± 5 bpm from the theoretical HRmax (208–0.7 × age) using a telemetry system (TM Fit, Hosand, Verbania, Italy) [26]. Starting from the covered distance in YYIRT1, an estimation of V’O_2_max was possible using the formula: estimate V’O_2_max (mlO_2_/kg/min) = YYIRT1 distance (m) × 0.0084 + 36.4 [27].

Lower-limb explosive power—Peak vertical power

Each player started the countermovement jump (CMJ) in the standing position, dropped into a self-selected depth on squat position and then immediately jumped as high as possible. The peak vertical power was measured with an inertial sensor (Gyko, Microgate, Bolzano, Italy) worn at the waist level. Each subject repeated the CMJ 3 times with a 10 seconds rest between trials [28]. The highest power was recorded.

### 2.5. Molecular Biology Analysis

Genomic DNA was extracted from buccal swab sample using the automated instrument MagCore Nucleic Acid Extractor (Diatech Pharmacogenetics, Jesi, Italy). The DNA concentration was determined using the NanoPhotometer P-Class (Implen, Munich, Germany). Mouth was rinsed with water before sampling. A buccal swab was collected for each subject. The samples were preserved at −20 °C temperature and processed within 72 h.

DNA sample was processed by polymerase chain reaction (PCR) using homemade primers (Table 1) designed using “Primers 3 software” (http://primer3.ut.ee/ accessed on 1 May 2018). The folding characteristic of PCR products and primers were determined using the secondary structure profiling software DINAMelt (http://unafold.rna.albany.edu/?q=mfold, accessed on 1 May 2018). Finally, BLAST software was used in order to test the specificity of primers sequences (http://blast.ncbi.nlm.nih.gov/Blast.cgi, accessed on 1 May 2018).

PCR was set up in 25 µL obtained by adding 12.5 µL of MasterMix 2X (Promega, Madison, WI, USA), containing Buffer (PH = 8.5), dNTPs (400 µM), Taq Polymerase (50 units/µL) and Mg+ (3 nM), 3 µL of genomic DNA as template (about 40–55 ng), 0.5 µL of primers (0.2 µM each) and ultrapure H_2_O until reaching the final volume. The PCR program was set on an initial denaturation at 95 °C for 2 min, followed by 35 cycles of 95 °C for 30 s, a step 30 s of Annealing Temperature conforming to the homemade primers, 72 °C for 30 s and a final extension at 72 °C for 5 min.

**Figure 2:**
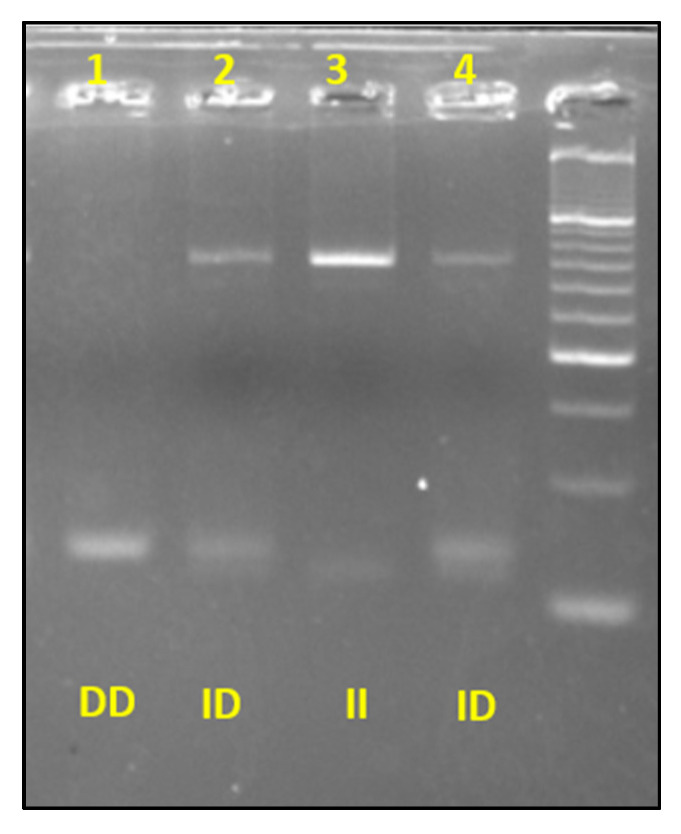
Electrophoretic profiles of three genotypes obtained by PCR of *ACE* I/D.

For rs4646994 *ACE* I/D variant, after the PCR step, total volume was loaded on a 4% agarose gel and subjected to electrophoresis for 30–35 min for *ACE* genotypes assignment according to the expected amplicon patterns (Figure 2). The genotypes’ results were analyzed and interpreted using the BioDoc-It ™ System UVP transilluminator.

**Figure 3:**
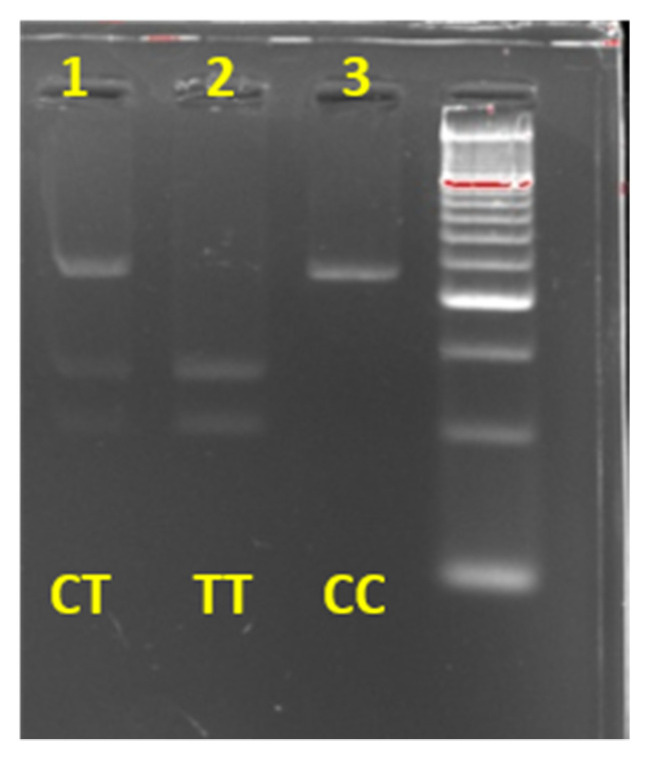
Electrophoretic profiles of genotypes obtained for *ACTN3*.

For rs1815739 *ACTN3* variant, PCR amplification products were loaded on an electrophoresis 2% agarose gel to verify the PCR efficiency. Following, an enzyme digestion was performed, adding 15 µL of PCR product, 0.5 µL of DdeI Restriction Enzyme associated with 2 µL of Buffer D (Promega, Madison, WI, USA) and 0.5 µL of BSA. Total volume was incubated at 37 °C for 3 h. After electrophoresis of 30–35 min on a 4% agarose gel, genotype assignment was performed using the BioDoc-It ™ System UVP transilluminator according to the expected amplicon patterns (Figure 3).

For rs1049434 *MCT1* variant, the Sanger sequencing was performed, in association to the Applied Biosystems 3500 Genetic Analyzer (Applied Biosystems, Foster City, CA, USA) according to manufacturer’s recommendation.

### 2.6. Statistical Analysis

Statistical analysis was performed by using the Statistical Package for Social Science (SPSS v.26). Continuous variables were expressed as mean ± standard deviation (SD), and categorical variables were displayed as frequencies. The unpaired t test was used to compare forwards and backs and between the dominant models. Effect size (ES) magnitude between groups was calculated using Hedges’ g with 95% confidence interval (CI), interpreted as trivial <0.20, small 0.20–0.59, moderate 0.60–1.19, large 1.20–1.99 [29]. The distribution of genotypes was determined by chi-square. To compare the athletes’ phenotypes between all genotypes, a one-way ANOVA was performed and Tukey–Kramer post hoc test was used to determine statistical differences among the genotype groups. The level of significance was set at *p* < 0.05.

## 3. Results

Players were divided by playing position: forwards and backs (*n* = 13 forwards and *n* = 14 backs). All players performed the tests with maximal effort. For V’O_2_max test, exhaustion was ensured in the presence of at least one of the previously described criteria. The average values for each parameter were: RPE 7.1 ± 1.9, La concentration 11.0 ± 2.0 mmol/l, HRmax 191.8 ± 8.1 bpm. No differences between backs and forwards were highlighted.

As showed in Table 2, considering anthropometrical characteristics, forwards are substantially heavier and markedly taller than backs. LMI was higher in forwards than backs. According with physiological characteristics, backs were more agile in the 505 agility test and faster in the 20 m sprint test; they had a lower total sprint time in RSA and a superior relative V’O_2_max. Moreover, peak vertical power was similar between the two groups with no significant difference.

**Figure 4:**
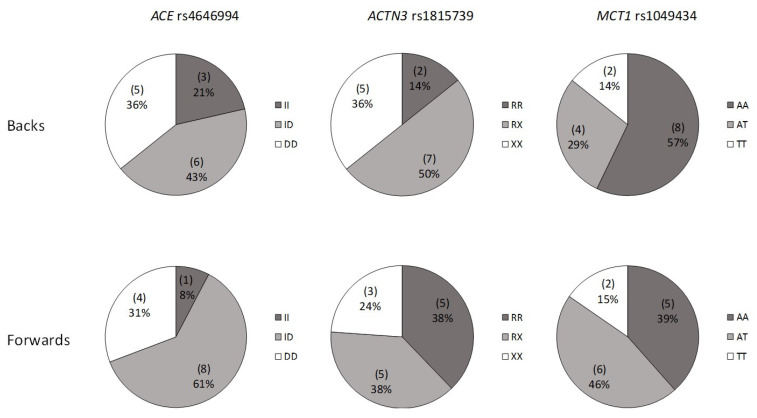
Genotype distribution in elite rugby union players for playing position.

**Figure 5:**
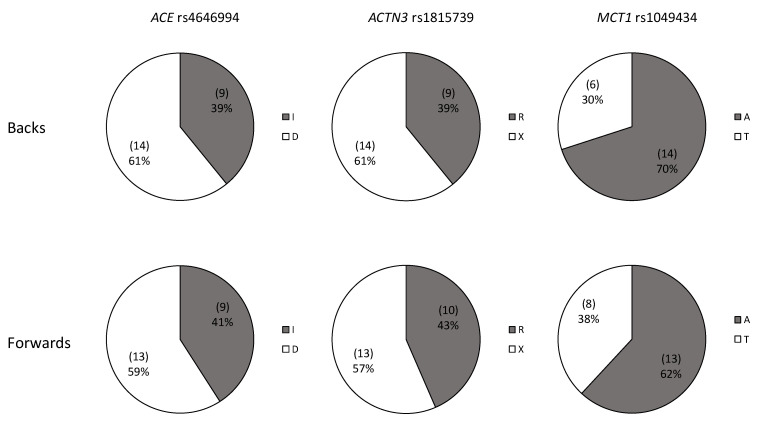
Allele distribution in elite rugby union players for playing position.

The genotype distribution was in Hardy–Weinberg equilibrium (*p* < 0.05). Genotypes and allele frequencies (Figure 4 and Figure 5) were comparable in absolute and relative values between backs and forwards (genotype chi-square, forwards vs. backs): *ACE*, χ^2^ = 1.362, *p* = 0.506; *ACTN3*, χ^2^ = 2.085, *p* = 0.353; *MCT1*, χ^2^ = 1.057, *p* = 0.590; allele frequency chi-square (forwards vs. backs): *ACE*, χ^2^ = 0.19, *p* = 0.890; *ACTN3*, χ^2^ = 0.626, *p* = 0.429; *MCT1*, χ^2^ = 0.320, *p* = 0.582).

*ACE* ID was the more frequently expressed genotype and proportional allele frequencies were greater in the D allele, in both groups. *ACTN3* RX genotype was the most represented in backs, but also in forwards together with the homozygous R genotype. Heterozygous AT genotype for *MCT1* has a greater proportion in forwards, while among the backs the AA genotype was more represented.

Table 3 and Table 4 outline the differences between genotypes according to different physical characteristics. Considering all phenotypes assessed, no significant differences between genotypes for both *ACE* I/D and *ACTN3* R577X were highlighted, even considering the I-dominant model (II + ID vs. DD) and R-dominant model (RR + RX vs. XX), respectively.

According to *MCT1* A1470T, the rugby players with the AA genotype were significantly faster in 505 agility test (*p* = 0.006, ES = 1.20) and 20 m sprint test (*p* = 0.012, ES = 1.15) than those carrying the AT genotype. Players with TT genotype expressed higher peak vertical power values than AT genotype (*p* = 0.037, ES = 1.30). Even the analysis of the T-dominant model (TT+AT vs. AA) confirmed that the players with the AA genotype were the fastest in the 505 agility test (*p* = 0.013, ES = 1.00) and in the 20 m sprint test (*p* = 0.017, ES = 0.95).

## 4. Discussion

Within the continuous evolution of sports to a higher professional level, a greater focus on details regarding the physical work for improving performance is now more important than ever. The need to turn to molecular biology derives from the fact that the ever-increasing requests for physical training personalization led to a mapping of the athlete not only at the phenotype level, but also at the molecular variants level that could influence the phenotype itself. The aim of the present study was to verify the modulation of genetic influence on rugby-specific physical performance. Our results showed three main findings: (1) forwards and backs were different both in anthropometrical and physiological characteristics; (2) there were no differences in genotype and allele frequencies in any polymorphisms; and (3) *MCT1* showed that the players with the AA genotype were the fastest both in the 505 agility and 20 m sprint tests.

Anthropometric and physical differences between forwards and backs have already been identified. Our study confirms that for anthropometric characteristics forwards are substantially heavier and markedly taller than backs [14] and LMI was higher in forwards than backs [25]. According with differences in physiological characteristics, backs were more agile and faster [30] and had a superior relative V’O_2_max [14]. Backs have outperformed forwards in leg power [23], even if the differences only approached significance in our study. Backs have also a superior ability to perform repeated sprint efforts challenging the anaerobic lactic metabolism [31].

*ACE* is a gene variant well recognized for endurance athlete status; furthermore, *ACE* and *ACTN3* are genetic markers well documented for power/strength athlete status [2]. *MCT1* was more recently associated with elite sprint/power athletic status [7]. Nonetheless, in RU the number of sport-specific papers is very limited and only three studies correlated both genotypes and phenotypes [21,22,23] and *MCT1* has not been investigated in RU thus far. Our study confirms no differences in genotype distribution and allele frequencies in *ACE* I/D polymorphisms in RU elite players [20,22]. Our data showed that, in accordance with Bell and Heffernan, the genotype representation for *ACE* was the same for forwards and for backs (ID > DD > II) [20,22]. The same results were found in allele frequency, where the D allele was more expressed than I [20,22]. *ACTN3* genotype distribution and allele frequencies, identified in the present study, are consistent only with the findings of Bell et al. [23], confirming no differences between forwards and backs in RU players. On the contrary, Heffernan et al. [20] highlighted that the X allele was overrepresented in forwards compared with backs. However, in all studies RX genotype and R allele were the most expressed in both forwards and backs [20,23]. Finally, our findings, while preliminary, suggest that genotype distribution and allele frequencies in *MCT1* were similar between forwards and backs. These findings seem to suggest that playing position is more linked to phenotype and training than to genotype.

This research found no genotype–phenotype association in *ACE* I/D polymorphisms. At the contrary Bell et al. [22] highlighted a greater leg power in University rugby players with ID genotype when compared with DD genotype. It could be speculated that a high volume of training performed by national level athletes would be able to reduce the genetic influence observed in lower levels athletes. Conversely, there are similarities in *ACTN3* R577X between the present study and those described by Bell [23]. In both studies no significant associations between genotypes and phenotypes expression were showed [23]. In other studies *ACTN3* R577X was associated with top-level sprint performance (specially the R allele) [32]. To the best of our knowledge, no investigations measured the influence of *MCT1* variants on RU athlete’s performance. *MCT1* analysis showed that the players with AA genotype were significantly faster than AT in 505 agility and in 20 m sprint tests, with a large and moderate effect size, respectively. Moreover, considering the T-dominant model for *MCT1*, the AA genotype remains significantly faster than TT+AT variants both in the 505 agility test and in the 20 m sprint test, with a moderate effect size. Furthermore, previous studies [5,14] suggested that the TT genotype characterized elite sprint/power athletic status; our data confirm this finding in peak vertical power, where TT genotype group reported significantly larger values than AT genotype, with a moderate effect size. These data must be interpreted with caution because of the small number of players in the TT group.

The lack of influence of *ACTN3* genotype on a variety of performance phenotypes has been already established in RU players [23]. Moreover, the importance of *ACE* I/D remains controversial in the literature [18]. On the contrary, there is a lack of information regarding the influence of the *MCT1* gene on team sport performance and especially in RU. Considering the key physiological factors, as highlighted by Brazier [16], an elite athlete must be agile and fast. Our findings suggest that the AA genotype in *MCT1* variants may confer an advantage both in agility and in speed. Considering that a combination between genetic profile and optimal training is an important factor for professional athletic performance, our results could have future implications such as the role of *MCT1* in training personalization for a better performance.

A strength of the study was the athletes’ level, with all athletes playing at professional and national or international level. All the subjects were elite RU players from the same team who competed in the same division, performing the same number of weekly training sessions and matches. Another strength was that three genes were analyzed, taking into consideration *MCT1* which was never investigated in RU players. Finally, a complete test battery was used to summarize specific qualities required in rugby. The research was clearly limited in the sample. It was not possible to involve a greater number of athletes from the entire roster, because the incidence of injury was relatively high during the competitive phase (16 players injured). The low number of subjects may somewhat explain the lack of observed effect. For this reason, a post hoc achieved power computation was performed (1 − β = 0.7).

## 5. Conclusions

Several genetic polymorphisms have been reported to be predictive of some aspects of athlete’s phenotype, providing useful information for coaches. In conclusion, in this study, the most predictive polymorphism seems to be the *MCT1*. This is the first study to demonstrate an association between the *MCT1* polymorphism and elite RU players’ phenotypes. Although further studies are required to elucidate its possible role, the *MCT1* polymorphism could be considered in the future to learn more about RU status. Future research should also concentrate on the investigation of genomics DNA profiling, anthropometric, and physical measurements combined to create tailored training programs to achieve optimal performance even in team sports, as suggested by some authors [33,34]. However, recent research must be taken in consideration to be aware about the difficult predictability of athletes’ level discrimination starting from genetic information [35].

## Figures and Tables

**Table 1:** Homemade primers sequence used for the DNA analysis.

Gene	Primers Sequence	Annealing Temperature for PCR
*ACE*	Forward F1, 5′-CCCATTTCTCTAGACCTGCT-3′ Forward F2, 5′-TGGGATTACAGGCGTGAT-3′ Reverse R1, 5′-AGAGCTGGAATAAAATTGGC-3′	55°
*ACTN3*	Forward, 5′-GGGCACACTGCTGCCCTTTC-3′ Reverse, 5′-GATGTCCTGCGGGCTGAG-3′	61°
*MCT1*	Forward, 5′-AGACCAGTATAGATGTTGCTGGG-3′ Reverse, 5′-CCACTGGTAGATTACAGGCCA-3′	58°

**Table 2:** Comparison of phenotypes for playing position in elite rugby union players.

	Forwards (*n* = 13)	Backs (*n* = 14)	*p*-Value	ES
Weight (kg)	106.7 ± 10.1	81.5 ± 9.6	<0.0001	2.48
Height (cm)	184.4 ± 5.5	177.4 ± 6.9	0.0055	1.14
LMI (kg/mm^0.14^)	55.1 ± 4.6	45.8 ± 4.8	<0.0001	1.93
Peak vertical power (W/kg)	69.5 ± 17.7	84.3 ± 23.6	0.978	0.01
505 agility test (s)	2.35 ± 0.16	2.18 ± 0.10	0.0032	1.22
20 m sprint test (s)	3.20 ± 0.16	2.95 ± 0.14	0.0002	1.62
RSA total sprint time (s)	41.94 ± 2.43	38.17 ± 0.93	<0.0001	2.01
V’O_2_max (mlO_2_/kg/min)	46.1 ± 3.2	51.1 ± 3.2	0.0004	1.52

Values are expressed in mean ± SD. Unpaired *t* test (forwards vs. backs). LMI, lean mass index; RSA, repeated-sprint ability; V’O_2_max, maximal oxygen consumption.

**Table 3:** ANOVA for physical characteristics of elite rugby union players; relative to *ACE*, *ACTN3* and *MCT1* genotypes.

	**Genotype**		
*ACE*	**II** **(*n* = 4)**	**ID** **(*n* = 14)**	**DD** **(*n* = 9)**
Physical characteristics			
LMI (kg/mm^0.14^)	54.4 ± 6.6	49.7 ± 6.7	49.5 ± 6.6
Peak vertical power (W/kg)	81.9 ± 26.7	76.9 ± 26.1	73.6 ± 19.1
505 agility test (s)	2.34 ± 0.30	2.25 ± 0.10	2.25 ± 0.16
20 m sprint (s)	3.12 ± 0.31	3.09 ± 0.15	3.03 ± 0.22
RSA total sprint time (s)	40.48 ± 4.28	40.13 ± 2.39	39.54 ± 2.39
V’O_2_max (mlO_2_/kg/min)	47.7 ± 4.6	48.9 ± 4.6	48.9 ± 3.0
	**Genotype**		
*ACTN3*	**RR**	**RX**	**XX**
Physical characteristics	**(*n* = 7)**	**(*n* = 12)**	**(*n* = 8)**
LMI (kg/mm^0.14^)	50.7 ± 5.7	50.5 ± 6.2	49.7 ± 8.6
Peak vertical power (W/kg)	78.2 ± 27.8	74.5 ± 17.6	78.2 ± 29.1
505 agility test (s)	2.27 ± 0.13	2.25 ± 0.13	2.28 ± 0.22
20 m sprint (s)	3.06 ± 0.20	3.07 ± 0.20	3.08 ± 0.21
RSA total sprint time (s)	40.10 ± 1.85	39.98 ± 2.88	39.88 ± 3.07
V’O_2_max (mlO_2_/kg/min)	48.3 ± 4.5	48.0 ± 3.6	50.1 ± 4.3
	**Genotype**		
*MCT1*	**AA**	**AT**	**TT**
Physical characteristics	**(*n* = 13)**	**(*n* = 10)**	**(*n* = 4)**
LMI (kg/mm^0.14^)	49.1 ± 7.1	52.0 ± 7.0	50.2 ± 3.4
Peak vertical power (W/kg)	78.6 ± 26.2	67.5 ± 19.1	92.6 ± 14.9 *
505 agility test (s)	2.19 ± 0.09 *	2.36 ± 0.19	2.26 ± 0.12
20 m sprint (s)	2.98 ± 0.17 *	3.19 ± 0.19	3.09 ± 0.19
RSA total sprint time (s)	39.23 ± 2.16	41.03 ± 3.28	39.82 ± 1.52
V’O_2_max (mlO_2_/kg/min)	41.3 ± 3.5	48.2 ± 5.1	48.9 ± 3.5

Values are expressed in mean ± SD. Tukey–Kramer post hoc test: * *p*-value < 0.05. *MCT1*: Peak vertical power, TT > AT; 505 agility test and 20 m sprint test, AA > AT.

**Table 4:** Physical characteristics of elite rugby union players; relative to *ACE*, *ACTN3*, and *MCT1* genotypes according to the dominant model.

	**Dominant Model**			
*ACE* Physical characteristics	**II + ID** **(*n* = 18)**	**DD** **(*n* = 9)**	***p*-Value**	**ES**
LMI (kg/mm^0.14^)	50.6 ± 6.6	49.5 ± 6.6	0.640	0.19
Peak vertical power (W/kg)	78.0 ± 25.5	73.6 ± 19.1	0.653	0.18
505 agility test (s)	2.27 ± 0.16	2.25 ± 0.16	0.714	0.15
20 m sprint (s)	3.09 ± 0.19	3.03 ± 0.22	0.446	0.31
RSA total sprint time (s)	40.21 ± 2.76	39.54 ± 2.39	0.542	0.25
V’O_2_max (mlO_2_/kg/min)	48.6 ± 4.5	48.9 ± 3.0	0.841	0.08
	**Dominant model**			
*ACTN3*	**RR + RX**	**XX**	***p*-Value**	**ES**
Physical characteristics	**(*n* = 19)**	**(*n* = 8)**		
LMI (kg/mm^0.14^)	50.6 ± 5.8	49.7 ± 8.6	0.767	0.12
Peak vertical power (W/kg)	75.9 ± 21.3	78.2 ± 29.1	0.820	0.09
505 agility test (s)	2.26 ± 0.13	2.28 ± 0.22	0.771	0.12
20 m sprint (s)	3.07 ± 0.19	3.08 ± 0.21	0.934	0.03
RSA total sprint time (s)	40.02 ± 2.49	39.88 ± 3.07	0.901	0.05
V’O_2_max (mlO_2_/kg/min)	48.1 ± 3.8	50.1 ± 4.3	0.242	0.49
	**Dominant model**			
*MCT1*	**TT + AT**	**AA**	***p*-Value**	**ES**
Physical characteristics	**(*n* = 14)**	**(*n* = 13)**		
LMI (kg/mm^0.14^)	51.4 ± 6.1	49.1 ± 7.1	0.359	0.35
Peak vertical power (W/kg)	74.6 ± 21.0	78.6 ± 26.2	0.668	0.16
505 agility test (s)	2.33 ± 0.17	2.19 ± 0.09	0.013	1.00
20 m sprint (s)	3.16 ± 0.19	2.98 ± 0.17	0.017	0.95
RSA total sprint time (s)	40.69 ± 2.88	39.23 ± 2.16	0.152	0.55
V’O_2_max (mlO_2_/kg/min)	48.4 ± 4.6	49.1 ± 3.5	0.641	0.18

Values are expressed in mean ± SD. Unpaired *t* test. *MCT1*: 505 agility test and 20 m sprint test, AA > TT + AT.

## Data Availability

The data presented in the study are available on request from the corresponding author due to restrictions (privacy).
